# Isolation and identification of ligninolytic bacterium (*Bacillus cereus*) from buffalo (*Bubalus bubalis*) rumen and its effects on the fermentation quality, nutrient composition, and bacterial community of rape silage

**DOI:** 10.3389/fmicb.2023.1103652

**Published:** 2023-04-18

**Authors:** Huimin Zhong, Jiayan Zhou, Fan Wang, Wenqing Wu, Haiqian Xiong, Huaihui Yin, Xiang Li

**Affiliations:** ^1^College of Life Science and Technology, Huazhong University of Science and Technology, Wuhan, China; ^2^CAS Key Laboratory of Quantitative Engineering Biology, Shenzhen Institute of Synthetic Biology, Shenzhen Institutes of Advanced Technology, Chinese Academy of Sciences, Shenzhen, China; ^3^National Center for International Research on Animal Genetics, Breeding and Reproduction (NCIRAGBR), Huazhong Agricultural University, Wuhan, China; ^4^Animal Husbandry, Huanggang Academy of Agricultural Sciences, Huanggang, Hubei, China; ^5^Key Laboratory of Agricultural Animal Genetics, Breeding and Reproduction of Ministry of Education, College of Animal Science and Technology, Huazhong Agricultural University, Wuhan, China; ^6^Shennongjia Science and Technology Innovation Center, Huazhong Agricultural University, Shennongjia, China

**Keywords:** *Bacillus cereus*, lignin-degrading, rape silage, buffalo rumen, microbial community

## Abstract

This study aimed to isolate and identify a ligninolytic bacterium from the rumen of buffalo (*Bubalus bubalis*) and investigate its effects as a silage additive for whole-plant rape. Three lignin-degradation strains were isolated from the buffalo rumen, with AH7-7 being chosen for further experiments. Strain AH7-7, with acid tolerance and a 51.4% survival rate at pH 4, was identified as *Bacillus cereus*. It exhibited a lignin-degradation rate of 20.5% after being inoculated in a lignin-degrading medium for 8 days. We divided the rape into four groups according to the various additive compositions to examine the fermentation quality, nutritional value, and bacterial community after ensiling: Bc group (inoculated with *B. cereus* AH7-7 3.0 × 10^6^ CFU g FW^−1^), Blac group (inoculated with *B. cereus* AH7-7 1.0 × 10^6^ CFU g FW^−1^, *L. plantarum* 1.0 × 10^6^ CFU g FW^−1^, and *L. buchneri* 1.0 × 10^6^ CFU g FW^−1^), Lac group (inoculated with *L. plantarum* 1.5 × 10^6^ CFU g FW^−1^ and *L. buchneri* 1.5 × 10^6^ CFU g FW^−1^), and Ctrl group (no additives). After 60 days of fermentation, the application of *B. cereus* AH7-7 was potent in modulating the fermentation quality of silage, especially when combined with *L. plantarum* and *L. buchneri*, as indicated by lower dry matter loss and higher contents of crude protein, water-soluble carbohydrate, and lactic acid. Furthermore, treatments with the *B. cereus* AH7-7 additive decreased the contents of acid detergent lignin, cellulose, and hemicellulose. The *B. cereus* AH7-7 additive treatments reduced the bacterial diversity and optimized the bacterial community compositions of silage, with an increase in the relative abundance of beneficial *Lactobacillus* and a decrease in the relative abundance of undesirable *Pantoea* and *Erwinia*. Functional prediction revealed that inoculation with *B. cereus* AH7-7 could increase the cofactors and vitamins metabolism, amino acid metabolism, translation, replication and repair, and nucleotide metabolism, while decreasing the carbohydrate metabolism, membrane transport, and energy metabolism. In brief, *B. cereus* AH7-7 improved the microbial community, fermentation activity, and ultimately the quality of silage. The ensiling with *B. cereus* AH7-7, *L. plantarum*, and *L. buchneri* combination is an effective and practical strategy to improve the fermentation and nutrition preservation of rape silage.

## 1. Introduction

The demand for animal products is rising in many developing nations, but the scarcity of forage is the major obstacle to the prosperity of animal husbandry (Sifeeldein et al., 2018). Due to the rising demand, it is crucial to create high-quality feed resources and effective preservation techniques. Rape silage is a kind of roughage with great development prospects that has a high metabolizable energy level (2.8–3.0 Mcal/kg DM) and a high crude protein level (160–200 g/kg DM) (Kaur et al., 2011; Barry, 2013). When the corn silage was partially replaced by rape silage, the apparent total-tract digestibility increased by 10.99% in dairy buffalo (Zhou et al., 2021).

Various types of additives have been used in silage to ensure appropriate fermentation and improve silage quality (Muck et al., 2018). These additives are chemical or microbiological, with microbiological additives being more prevalent (Muck et al., 2018). Chemical additives are inferior to microbiological additives because microbiological additives are naturally occurring, do not cause environmental harm, and do not corrode machinery (Filya, 2003). Lactic acid bacteria are the main bacterial group as inoculants in silage and have been shown to produce lactic acid that is beneficial for silage preservation (Muck et al., 2018; Fabiszewska et al., 2019). *L. plantarum, L. acidophilus, Enterococcus faecium, P. pentosaceus*, and *Pediococcus acidilactici* are the principal species used in silage to rapidly increase lactic acid and decrease pH (Muck et al., 2018; Fabiszewska et al., 2019). Due to the antifungal effects of acetic and propionic acid, *L. buchneri* is the main species used as an inoculant to improve aerobic stability (Krooneman et al., 2002; da Silva et al., 2018). In the form of one or more species of microorganisms, the inoculants have varying effects on the silage (Carvalho et al., 2021). The use of these inoculants in silage reduces DM loss, increases the yield of microbial metabolites of interest, inhibits undesirable microorganisms, and improves microbial and nutrient quality (Oliveira et al., 2017; Muck et al., 2018). Correct selection and application of inoculants are essential for obtaining high-quality silage. Many unknown microorganisms that can play an important role in silage are worth exploring.

Lignin, an important component of roughage that is embedded between cellulose and hemicellulose in plants, affects the nutritional value and utilization rate of roughage and hinders the digestion ability of ordinary animals (Zakzeski et al., 2010). Buffaloes live in the arid tropics, which are different from other ruminants. Buffaloes were found to have higher digestibilities of DM, crude protein, and organic matter in rice straw than cattle (Terramoccia et al., 2000; Chanthakhoun et al., 2012). Interestingly, buffalo was the first mammal to be verified to have the ability of lignin degradation (Xu et al., 2021). According to a prior study, strains with lignin degradation ability were isolated from buffalo rumen, and the degradation rate of sodium lignosulfonate could reach 11.1% (Wang et al., 2021). This suggests that rumen microbes play an important role in the buffalo's ability to exhibit lignin degradation. Rumen microorganisms were frequently used in silage research to investigate their effects on silage. To accelerate silage fermentation, anaerobic fungi from goat rumen were introduced into the rice straw silage (Lee et al., 2015). To improve the quality of the silage, the fibrolytic cellulolytic fungi and bacteria from yak rumen were applied to *Pennisetum sinese* silage (Li et al., 2018). The uniqueness of buffalo rumen microorganisms leads to their potential application in silage.

The rape production area is the main distribution area of the buffalo population. However, there is no research on inoculating lignin-degrading bacteria from buffalo rumen in rape silage to improve the quality. Therefore, research into the association between the buffalo ruminal microbiota and rape silage quality is of great scientific and commercial value. The present study aimed to explore the potential of *B. cereus* AH7-7 as an additive in silage. We hypothesized that the *B. cereus* AH7-7 inoculum inoculated in rape silage could regulate fermentation quality, nutrient composition, and the bacterial community of rape silage. Our findings might shed light on the use of bacteria from the buffalo rumen to improve the fermentation quality of rape silage.

## 2. Materials and methods

### 2.1. Isolation of lignin-degrading bacteria

The ruminal fluid collection was approved by the Animal Experimental Ethical Inspection of Laboratory Animal Center, Huazhong Agriculture University (HZAUCA-2018-003). This experiment is in line with the national regulations regarding animal welfare ethics.

As inoculum material, the rumen content of three fistula buffalo rumens (Mediterranean Nili-Ravi, 7 years old, 572 ± 24 kg) was mixed. These animals were fed at Jinniu Animal Husbandry Co., Ltd (Hubei, China). To avoid forage disturbance, all animals fasted for 24 h before collection. The inoculum was inoculated into the enrichment medium for 3 days before being carefully transferred to the lignin-degrading screening medium; both of these operations were performed at 39°C under anaerobic conditions. We performed three consecutive transfer screens over 10 days using screening media, and then the final screening cultures were diluted with a normal saline gradient and scribed on Luria broth agar to obtain different kinds of colonies. The lignin-degradation experiment was performed in a 250 ml flask with 100 ml of sodium ligninsulfonate in a mineral salt medium with a pH of 6.5 (Raj et al., 2007; Wang et al., 2013). The concentration of sodium ligninsulfonate in the degradation medium was 1.0 mg/L. The bacterial culture medium with a concentration of 10^6^ CFU/ml was inoculated into three duplicate flasks, each of which was inoculated with 1 ml. The control medium was uninoculated sodium ligninsulfonate containing mineral salt. The flasks were cultured on a rotary shaker at 39°C, 90×*g* under aerobic conditions for 8 days. We extracted samples from the flask regularly once a day and determined the content of sodium ligninsulfonate. After centrifugation at 6,000×g for 10 min, the supernatant fluids of the treatment and control groups were both acidified to pH 1–2 using 12 M HCl to get the precipitate. Then the precipitate was gathered after centrifugation at 9,500 × *g* for 10 mins. Residual sodium ligninsulfonate was obtained after the precipitate was washed with deionized water and dried at 65°C for 2 days to constant weight.

The compositions of the culture medium were as follows:

Buffer A (phosphate-buffered mineral salts medium A, per liter): [0.4 g CaCl_2_·2H_2_O; 3.0 g (NH_4_)_2_SO_4_; 0.6 g MgSO_4_·7H_2_O; 3.0 g KH_2_PO_4_; 6.0 g NaCl]. Buffer B (phosphate-buffered mineral salts medium B, per liter): [4.0 g K_2_HPO_4_·3H_2_O]. Rumen fluid: removed solid rumen contents by filtration with four layers of repeated gauze, centrifuged at 6,500 × *g* under 4°C for 10 min, and then the supernatant fluid of three buffalo was mixed in the same proportion to gain rumen fluid.

The enrichment medium (per liter): 170 ml of rumen fluid, 165 ml of phosphate-buffered mineral salts medium A, 165 ml of phosphate-buffered mineral salts medium B, 0.5 mg of copper sulfate, 1.0 g of tryptone, and 1.0 g of yeast extract. The screening medium (per liter): 165 ml of phosphate-buffered mineral salts medium A, 165 ml of phosphate-buffered mineral salts medium B, and 5.0 g of sodium lignosulfonate.

**Figure 1:**
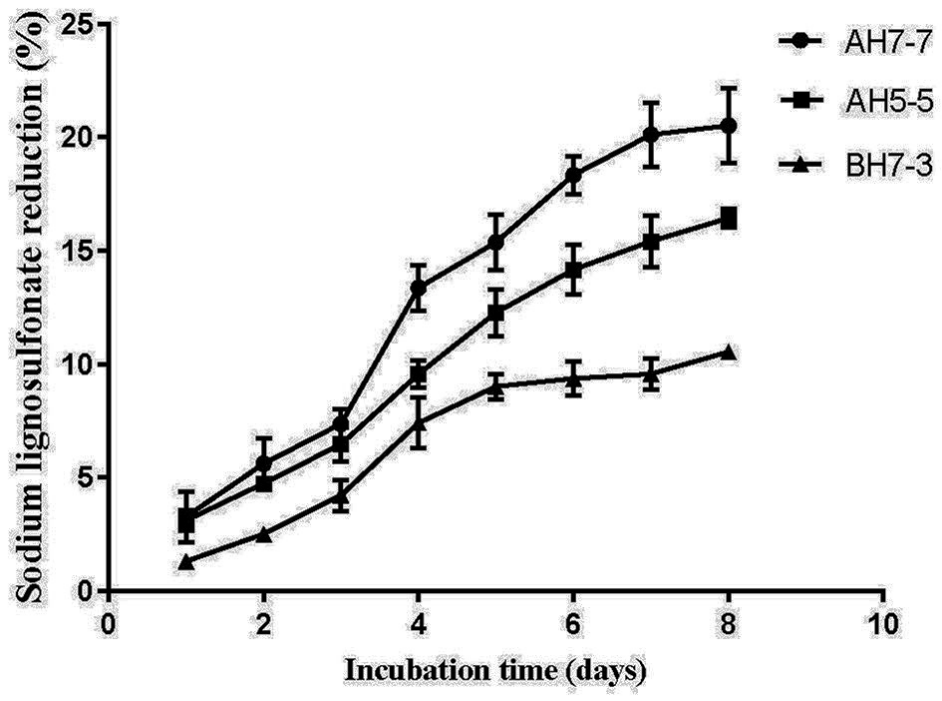
Sodium ligninsulfonate reduction by three strains.

Three different strains with lignin degradation ability were isolated from the rumen of buffalo, namely, AH5-5, AH7-7, and BH7-3. The results of lignin degradation by three strains showed that AH7-7 had the strongest lignin degradation ability (Figure 1). We selected the strain AH7-7 for subsequent silage experiments.

### 2.2. Identification of strain AH7-7

The purified strain AH7-7 was successively diluted with sterile saline, streaked onto the Luria Broth agar, and incubated at 39°C for 24 h, after which the characteristics of colonies were observed. The isolated strain AH7-7 was observed by Gram staining. The acid resistance of the strain AH7-7 was measured according to the previous method (Wu et al., 2014). Classical physiological and biochemical characteristics of the strain AH7-7 were tentatively identified according to the Taxonomic Outline of the Prokaryotes Bergey's Manual of Systematic Bacteriology (Garrity et al., 2004).

Bacterial 16S rRNA gene sequencing and gyrB gene sequencing based on Sanger sequencing were applied for molecular identification (Zhong et al., 2022). For amplification in 16S rRNA gene sequencing, the forward primer was 27F (5′-AGAGTTTGATCCTGGCTCAG-3′), and the reverse primer was 1492R (5′-GGTTACCTTGTTACGACTT-3′). In gyrB gene sequencing, the forward primer was gyrbF (5′-ATTGGTGACACCGATCAAACA-3′), and the reverse primer was gyrbR (5′-TCATACGTATGGATGTTATTC-3′). The total reaction volume was 30 μl, which contained 10.5 μl of nuclease-free water, 1.5 μl of genomic DNA, 1.5 μl of each primer, and 15 μl of Master Mix (KOD ONE MM, TOYOBO). The PCR setting was as follows: initial denaturation at 95°C for 2 min, followed by 25 cycles (denaturation at 98°C for 10 s, annealing at 55°C for 30 s, extension at 72°C for 90 s), and the final extension at 72°C for 2 min. The figure of the phylogenetic tree was drawn by MEGA7 software using the maximum likelihood statistical method. Then the phylogeny was tested by the bootstrap method; the number of bootstrap replications was 1,000.

### 2.3. Preparation of rape silage

In this experiment, the whole rape (*Brassica napus*), harvested from the rape experimental base of the Xianning Academy of Agricultural Sciences in Hubei Province, was used as the substrate for silage production. A total of 18,100 m^2^ plots of rape were cultivated in the experimental field of the Xianning Academy of Agricultural Sciences (N 29°50′, E 114°19′). This area has a subtropical continental monsoon climate with an average temperature of 16.8°C, an average elevation of 189.6 m, and a mean annual precipitation of 1,523.3 mm. To eliminate unnecessary experimental errors, we randomly selected six rapeseed plots, which produced enough rape biomass to be required for this experiment. The whole rape was sown at a 0.35 kg/mu seed rate. Rape was harvested at the peak blooming stage, wilted in the field, tedded every 2.0 h for 6 h (the DM content was 329.20 g kg^−1^ after wilting), and carried to the laboratory. The chemical and microbial compositions of fresh wilted rape are shown in Table 1.

**Table 1:** Chemical composition and microbial population before ensiling.

**Chemical composition**	**FM**
Dry matter (g/kg)	329.20
Crude protein (g/kg DM)	122.97
Water soluble carbohydrates (g/kg DM)	72.43
Neutral detergent fiber (g/kg DM)	543.49
Acid detergent fiber (g/kg DM)	376.19
Acid detergent lignin (g/kg DM)	63.65
Cellulose (g/kg DM)	323.18
Hemicellulose (g/kg DM)	70.45
**Microbial population (log**_10_ **CFU/g of FM)**	
Lactic acid bacteria	5.26
Yeasts	4.37
Molds	ND
*Enterobacter*	3.56

FM, fresh material of silage; ND, not detected; CFU, colony-forming unit.

The wilted rape was cut into 1–3 cm long pieces with a cutter, mixed thoroughly with additives, and packed into a sterile plastic bag (25 × 30 cm). The bags were compacted and vacuum-sealed by a vacuum sealer machine. Depending on the additives, silage was divided into four treatment groups: Bc group (inoculated with *B. cereus* AH7-7 3.0 × 10^6^ CFU g FW^−1^), Blac group (inoculated with *B. cereus* AH7-7 1.0 × 10^6^ CFU g FW^−1^, *L. plantarum* 1.0 × 10^6^ CFU g FW^−1^, and *L. buchneri* 1.0 × 10^6^ CFU g FW^−1^), Lac group (inoculated with *L. plantarum* 1.5 × 10^6^ CFU g FW^−1^ and *L. buchneri* 1.5 × 10^6^ CFU g FW^−1^), and Ctrl group (no additives). In addition to the buffalo rumen strain AH7-7, the additives used in rape silage fermentation were *L. plantarum* (strain model: Lp90) and *L. buchneri* (strain model: LBu01), which were purchased from Jiangsu Weikang Biotechnology Co., Ltd. All groups of silage were conserved at ambient temperature (25–30°C), and ensiling was performed in quadruple repetitions. The bags were opened after 10, 30, and 60 days of ensiling for the analysis of the fermentation parameters and bacterial community.

### 2.4. Determination of chemical compositions and fermentation characteristics of rape silage

At the end of 60 days of ensiling, the whole contents of each group were mixed uniformly in a clean plastic container (Wagner, 2015). Three duplicate samples from each group were collected to determine the chemical composition and fermentation characteristics of rape silage. In total, 3 g of rape silage material was extracted with 27 ml of neutral distilled water for 12 h, and the pH value of the rape silage was determined by a pH meter. A total of 10 g of silage was soaked in deionized water at 4°C for 12 h to determine the contents of NH_3_-N and lactic acid. The content of NH_3_-N was measured by phenol-sodium hypochlorite colorimetry (Weatherburn, 1967). In total, 1.0 ml of silage's filtrate, 2.5 ml of phenol solution, and 2.0 ml of alkaline sodium hypochlorite solution were mixed, kept in a water bath at 37°C for 10 min, and the NH_3_-N content was measured at λ = 650 nm. The content of lactic acid in the silage was determined by p-hydroxybiphenyl colorimetry (Taylor, 1996). After mixing 0.5 ml of silage's filtrate with 0.05 ml of 4% copper sulfate pentahydrate, 6 ml of 98% H_2_SO_4_ was added and kept in boiling water for 5 min, and 2 ml of p-hydroxybenzene was added after cooling. The above solutions were mixed evenly, kept in the water bath at 30°C for 0.5 h, taken out, kept in boiling water for 90 s, and the lactic acid content was measured at λ = 560 nm after cooling. The samples of silage's filtrate from each group were centrifuged at 12,000 rpm for 10 min and then passed through a 0.22 μm filter. Then, acetic acid, propionic acid, and butyric acid were analyzed by high-phase liquid chromatography (U3000, Thermo Fisher Scientific, USA). The wavelength was 210 nm. The mobile phase was 3 mmol/L of perchloric acid, with a column temperature of 50°C, and the flow rate was 0.5 ml/min.

A total of 100 g samples were dried at 65°C for at least 48 h to a constant weight to determine the dry matter (DM) content and then ground by a 1.00 mm sieve for the following analysis (Udén et al., 2005). The crude protein (CP), neutral detergent fiber (NDF), acid detergent fiber (ADF), and acid detergent lignin (ADL) of rape silage and whole-plant rape were measured by the methods formulated by the Association of Official Analytical Chemists (AOAC) (Udén et al., 2005). The total nitrogen (TN) content was determined by the Kjeldahl procedure (Krishnamoorthy et al., 1982). The content of water-soluble carbohydrates (WSC) was determined by anthrone colorimetry (Leng et al., 2016). The chemical compositions of fresh rape material were also performed as the abovementioned methods.

### 2.5. DNA extraction and sequencing of microbial diversity

At the end of 60 days of ensiling, five duplicate samples from each group were collected for microbial sequencing. The samples were centrifuged at 10,000 *g* for 15 min to produce particles for subsequent DNA extraction. The microbial DNA in silage was extracted according to the PowerSoil^®^ DNA Isolation Kit (Mobio, San Diego, CA, USA) (Zhao et al., 2022). The bacterial 16S rDNA genes were amplified by PCR, using the 27F (5′-AGRGTTTGATYNTGGCTCAG-3′) as the forward primer and the 1492R (5′-TASGGHTACCTTGTTASGACTT-3′) as the reverse primer. PCR amplification was performed in a total reaction volume of 30 μl, which contained 15 μl of KOD One^TM^ PCR Master Mix (TOYOBO, China), 1.5 μl of each primer with a barcode, 1.5 μl of genomic DNA, and 10.5 μl of nuclease-free water. The PCR setup was as follows: initial denaturation at 95°C for 5 min, followed by 30 cycles (denaturation at 95°C for 30 s, annealing at 50°C for 30 s, extension at 72°C for 1 min), and final extension at 72°C for 7 min. The PCR products were purified (MagicPure^®^ Size Selection DNA Beads), quantified, and homogenized to obtain the SMRTbell sequence library. The library was sequenced on the PacBio platform after passing the quality inspection, using a single-molecule real-time sequencing method. Biomarker Technologies Corporation (Beijing, China) completed all the procedures mentioned above.

After completing PacBio platform sequencing, the data were exported to circular consensus sequencing (CCS) file, and then the CCS was identified by lima v1.7.0 software through barcode to obtain raw-CCS. The primer sequences of raw-CCS data were identified and removed by cutadapt 1.9.1 software. Raw-CCS was filtered to obtain clean-CCS data according to the sequence length. After identifying and removing the chimeric sequence of clean-CCS using UCHIME v.8.1 software, the effective-CCS was obtained (Edgar et al., 2011). The USEARCH v.10.0 software was adopted to divide the effective-CCS into different clusters of operational taxonomic units (OTUs) according to 97% similarity (Edgar, 2013). After that, the OTU sequences were annotated in SILVA bacterial 16S rRNA database (Release132, http://www.arb-silva.de) (Quast et al., 2013). The detected microbial communities were identified and annotated at various taxonomic levels. All the sample species were normalized (absolute abundance of species in the sample/total number of sequences sequenced in the sample).

### 2.6. Microbial population counting at 10, 30, and 60 days after ensiling

After 10, 30, and 60 days of ensiling, the equal contents of each group were taken out and mixed uniformly in a clean plastic container. Three duplicate samples from each group were collected to count the microbial population. The microbial population was counted as previously reported (Zhao et al., 2022). In total, 10 g of samples were mixed with 90 ml of sterile saline (0.85% NaCl). The mixture solutions were serially diluted from 10^−1^ to 10^−7^. The diluted solutions were coated on the surface of the medium plates. De Man, Rogosa, and Sharpe agar (Qingdao Hope Bio-Technology Co., Ltd.) were used to identify and count the lactic acid bacteria after anaerobic growth at 37°C for 48 h. Potato dextrose agar (Difco, Hopebil, Qingdao, China) were used to detect yeasts and molds and counted after aerobic growth at 28°C for 72 h. The microbial population counting of fresh rape material is also performed using the abovementioned methods.

### 2.7. Health observation and measurement

Blood samples from buffaloes were collected from the jugular vein using an EDTA blood collector (produced by Winner Medical Co, Ltd) for routine blood analysis before feeding. Blood was stored at 4°C and measured by the automatic blood cell analyzer (SF-3000, Sysmex-Toa Medical Electronics, Kobe, Japan). Body conditions were scored according to the previous standard (Hut et al., 2021). The DM feed intake of the buffaloes was calculated 1 month before the separation of strains in the rumen.

### 2.8. Statistical analysis

Data on nutritional components and basic characteristics of rape silage were calculated and statistically analyzed by the SPSS software version 19.0 (SPSS, Inc., Chicago, IL, United States). The data were normally distributed and homoscedastic. The yeast level after 10 days of ensiling was analyzed by an unpaired *t*-test. The lactic acid bacteria level after 10, 30, and 60 days of ensiling was analyzed using a statistical mixed model (repeated measures). Fixed effects include treatment groups, measurement times, and the interaction between treatment groups and measurement time. Random effects include covariance between repeated measures in samples. The rest data on nutritional components of rape silage were analyzed by one-way ANOVA. In the case of significant differences, the Duncan method was used for pairwise comparison. All data were expressed as means and standard errors of means (SEM).

Alpha diversity analysis, beta diversity analysis, and line discriminant analysis (LDA) effect size analysis were applied to analyze the microbial diversity. The alpha diversity index of the silage samples was evaluated by using the software mothur (version v.1.30). GraphPad Prism 7 was used for the box plots, and after testing the normality and homogeneity, all data were analyzed by one-way ANOVA. Microbial community structure was examined using principal component analysis (PCA), and groups were compared using an unweighted UniFrac distance matrix. The LDA threshold was set to >4.0, and the classification level is from phylum to species. Functional gene prediction based on the Kyoto Encyclopedia of Genes and Genomes (KEGG) databases was conducted using PICRUSt2 according to a previous report (Langille et al., 2013). The function level was level 2, and the *P* < 0.05 was considered a significant difference, and *P* < 0.01 was considered an extremely significant difference.

## 3. Results

### 3.1. Identification of the strain AH7-7

The result showed that the strain AH7-7 had a higher reduction of sodium ligninsulfonate content than strains AH5-5 and BH7-3 (Figure 1). The reduction in sodium ligninsulfonate content (20.5%) was observed after 8 days of incubation in AH7-7.

**Figure 2:**
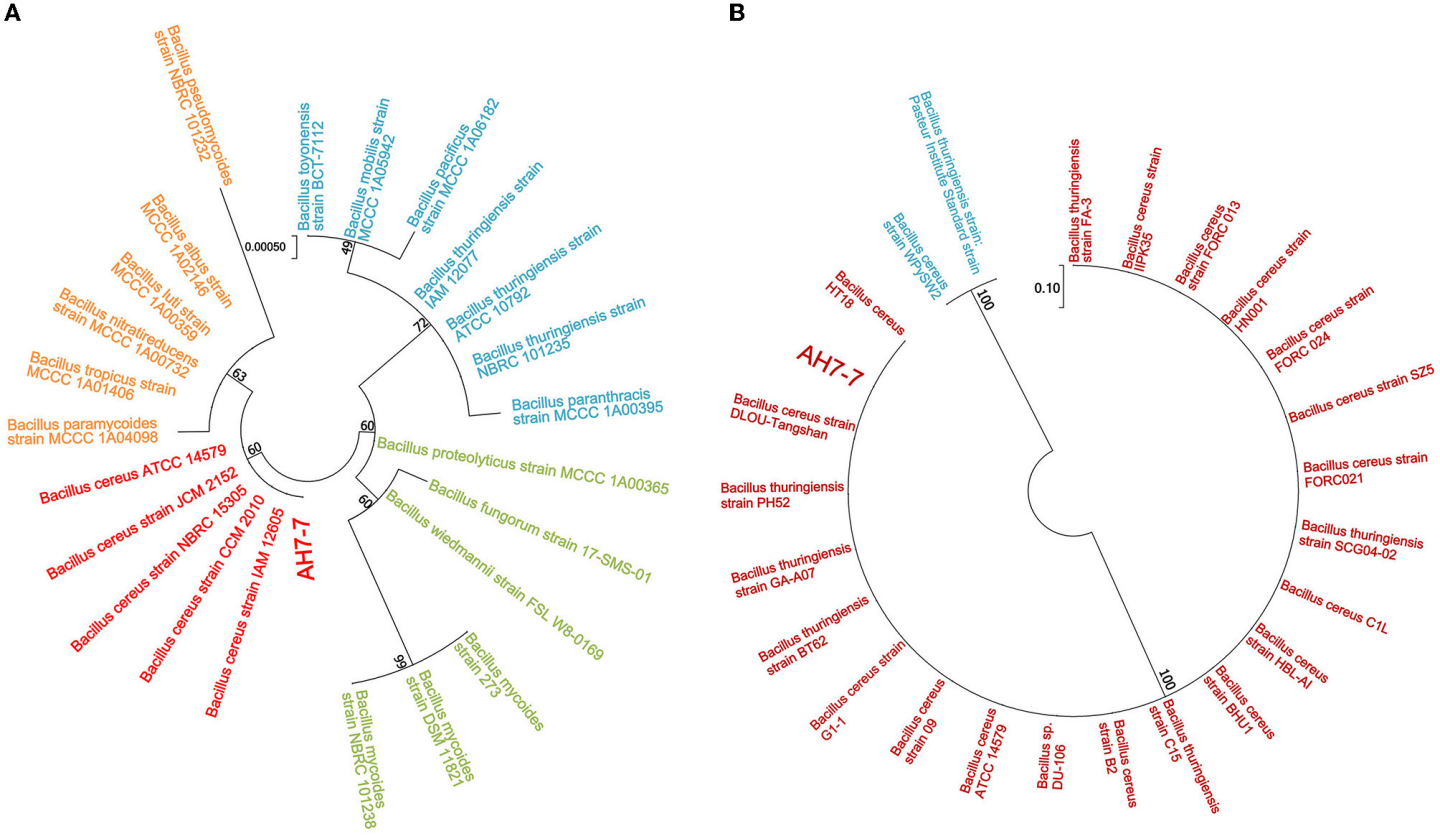
Phylogenetic tree of strain AH7-7. **(A)** 16S rRNA and **(B)** gyrB.

The colonies of the strain AH7-7 were large, waxy, round, translucent, and grayish white, with a rough surface and irregular edges (Supplementary Figure 1). The strain was identified as gram-positive according to Gram staining observed using the microscope (Supplementary Figure 1). The results of the acid tolerance test showed that the survival rates of the strain AH7-7 at pH = 2, 3, 4, and 5 were 13.0%, 32.9%, 51.4%, and 86.1%, respectively (Supplementary Figure 2). The analysis of the similarity of the 16S rRNA and gyrB nucleotide sequences demonstrated that the strain AH7-7 was the closest to *B. cereus* (Figure 2). The results of the physiological and biochemical characteristics of the strain AH7-7 are shown in Supplementary Tables 1, 2. According to colony observation, Gram staining, 16S rRNA gene sequencing, gyrB gene sequencing, and physiological and biochemical data, the strain AH7-7 was identified as *B. cereus*. These results are referenced from the literature: “Whole-Genome Sequencing Reveals Lignin-Degrading Capacity of a Ligninolytic Bacterium (*B. cereus*) from Buffalo (*Bubalus bubalis*) Rumen” (Zhong et al., 2022).

### 3.2. Effects of *B. cereus* AH7-7 on buffalo

The results of the blood routine, dry matter (DM) feed intake, and body condition scores are shown in Supplementary Table 3. All the tests of the blood routine were within the normal range. The buffaloes had a healthy body condition, and their feed intake was normal.

### 3.3. Initial characteristics of fresh rape

The chemical composition and microbial population of fresh rape are shown in Table 1. The contents of DM and WSC in rape were 329.2 g/kg FM and 72.43 g/kg DM, respectively. Rape contained crude protein, neutral detergent fiber, acid detergent fiber, acid detergent lignin, cellulose, and hemicellulose at 122.97, 543.49, 376.19, 63.65, 323.18, and 70.45 g/kg DM, respectively. The lactic acid bacteria (LAB), yeasts, and *Enterobacter* numbers of rape were 5.26, 4.37, and 3.56 log_10_ CFU/g FM.

### 3.4. The microbial population of rape silage on days 10, 30, and 60 ensiling

The microbial population at 10, 30, and 60 ensiling days is shown in Table 2. The LAB population increased with the fermentation time and was the highest on day 60 (*P* < 0.01). Compared to the Ctrl which had no additive, the number of LAB in the Bc group, which was inoculated with *B. cereus* AH7-7 alone, and the Blac group, which was applied the combination of *B. cereus* AH7-7, *L. plantarum*, and *L. buchneri*, was significantly higher (*P* < 0.01). No molds or *Enterobacter* were detected in silage for all fermentation days. The yeasts were detected on day 10 after the ensiling of the Lac group which was inoculated with the combination of *L. plantarum* and *L. buchneri* and the Ctrl group, while they disappeared after 30 and 60 days of silage, respectively.

**Table 2:** Microbial population after 10, 30, and 60 days ensiling.

**Items**	**Microbial population (log**_**10**_ **CFU/g of FM)**				**SEM**	***P*** **-value**		
	**Bc**	**Blac**	**Ctrl**	**Lac**		**G**	**T**	**G**×**T**
Lactic acid bacteria, 10 days	7.32^a^	7.45^a^	6.80^b^	7.56^a^	0.08	<0.01	<0.01	0.61
Lactic acid bacteria, 30 days	8.54^a^	8.63^a^	7.95^b^	8.56^a^	0.05	–	–	–
Lactic acid bacteria, 60 days	8.69^a^	8.88^a^	8.04^b^	8.67^a^	0.07	–	–	–
Yeasts, 10 days	ND	ND	5.13^a^	4.00^b^	0.20	<0.01		
Yeasts, 30/60 days	ND	ND	ND	ND	–	–		
Molds, 10/30/60 days	ND	ND	ND	ND	–	–		
*Enterobacter*, 10/30/60 days	ND	ND	ND	ND	–	–		

Different letters within the same line indicate statistically significant differences between the treatments (P < 0.05); Bc, added with strain *B. cereus* AH7-7; Blac, added with strain *B. cereus* AH7-7, *Lactobacillus plantarum*, and *Lactobacillus buchneri*; Lac, added with *Lactobacillus plantarum* and *Lactobacillus buchneri*; Ctrl, no additives; FM, fresh material of silage; SEM, standard error of mean; ND, not detected; CFU, colony-forming unit; G, effect of treatment groups; T, effect of measurement time; G × T, interaction between treatment groups and measurement time.

### 3.5. Fermentation characteristics of rape silage after 60 days of ensiling

The fermentation parameters of rape silages were different among all treatments (Table 3). Compared to the Ctrl group, all additive treatments (Bc, Blac, and Lac) decreased the pH values and increased the lactic acid, acetic acid, and propionic acid contents (*P* < 0.01). The lactic acid content was highest in the Blac group. Acetic acid content and propionic acid content were higher in the groups (Bc and Blac) with *B. cereus* AH7-7 additive. All additive treatments (Bc, Blac, and Lac) decreased NH_3_-N and butyric acid contents compared with the Ctrl group (*P* < 0.01).

**Table 3:** Fermentation characteristics of rape silage after 60 days ensiling.

**Items**	**Bc**	**Blac**	**Ctrl**	**Lac**	**SEM**	***P*** **-value**
pH	4.04^a^	4.06^a^	4.28^b^	4.09^a^	0.03	<0.01
NH_3_-N/ TN (%)	4.44^a^	4.74^a^	6.16^b^	4.96^a^	0.21	<0.01
Lactic acid (g/kg DM)	51.73^a^	57.13^b^	42.80^c^	49.05^a^	1.59	<0.01
Acetic acid (g/kg DM)	24.47^a^	24.79^a^	12.39^b^	17.71^c^	1.61	<0.01
Butyric acid (g/kg DM)	0.51^a^	0.42^a^	1.29^b^	0.46^a^	0.11	<0.01
Propionic acid (g/kg DM)	2.46^a^	2.50^a^	0.77^b^	1.24^c^	0.23	<0.01

Different letters within the same line indicate statistically significant differences between the treatments (P < 0.05); Bc, added with strain *B. cereus* AH7-7; Blac, added with strain *B. cereus* AH7-7, *Lactobacillus plantarum*, and *Lactobacillus buchneri*; Lac, added with *Lactobacillus plantarum* and *Lactobacillus buchneri*; Ctrl, no additives; NH_3_-N/TN, ammonium nitrogen/total nitrogen; DM, dry matter; SEM, standard error of mean.

### 3.6. Chemical composition of rape silage after 60 days of ensiling

As shown in Table 4, the DM of all groups ranged from 321.32 to 339.29 g/kg, with the Ctrl group having the lowest (321.32 g/kg, *P* < 0.01). Compared with the Lac and Ctrl groups, the contents of CP and WSC were affected by the *B. cereus* AH7-7 additive. The contents of CP and WSC were higher in the Bc and Blac groups (*P* < 0.01). The WSC content was highest in the Blac group (*P* < 0.01). Compared with the groups without the *B. cereus* AH7-7 additive (Ctrl and Lac), the contents of acid detergent lignin, cellulose, and hemicellulose in the Bc and Blac groups were significantly lower (*P* < 0.01).

**Table 4:** Chemical composition of rape silage after 60 days ensiling.

**Items**	**Bc**	**Blac**	**Ctrl**	**Lac**	**SEM**	***P*** **-value**
Dry matter (g/kg)	330.22^a^	339.29^c^	321.32^b^	332.44^a^	2.02	<0.01
Crude protein (g/kg DM)	131.13^a^	128.55^a^	106.08^b^	115.58^c^	2.39	<0.01
Water soluble carbohydrate (g/kg DM)	25.79^a^	28.86^b^	19.31^c^	23.24^a^	1.08	<0.01
Neutral detergent fiber (g/kg DM)	500.47	504.13	520.69	536.21	6.21	0.15
Acid detergent fiber (g/kg DM)	337.64^a^	332.11^a^	360.30^b^	367.60^b^	5.38	<0.05
Acid detergent lignin (g/kg DM)	55.78^a^	53.77^a^	63.92^b^	64.13^b^	1.29	<0.01
Cellulose (g/kg DM)	281.16^a^	272.86^a^	310.31^b^	309.46^b^	5.23	<0.01
Hemicellulose (g/kg DM)	50.03^a^	51.02^a^	60.67^b^	57.88^b^	1.30	<0.01

Different letters within the same line indicate statistically significant differences between the treatments (P < 0.05); Bc, added with strain *B. cereus* AH7-7; Blac, added with strain *B. cereus* AH7-7, *Lactobacillus plantarum*, and *Lactobacillus buchneri*; Lac, added with *Lactobacillus plantarum* and *Lactobacillus buchneri*; Ctrl, no additives; DM, dry matter; SEM, standard error of mean.

### 3.7. Microbial community of rape silage after 60 days of ensiling

**Figure 3:**
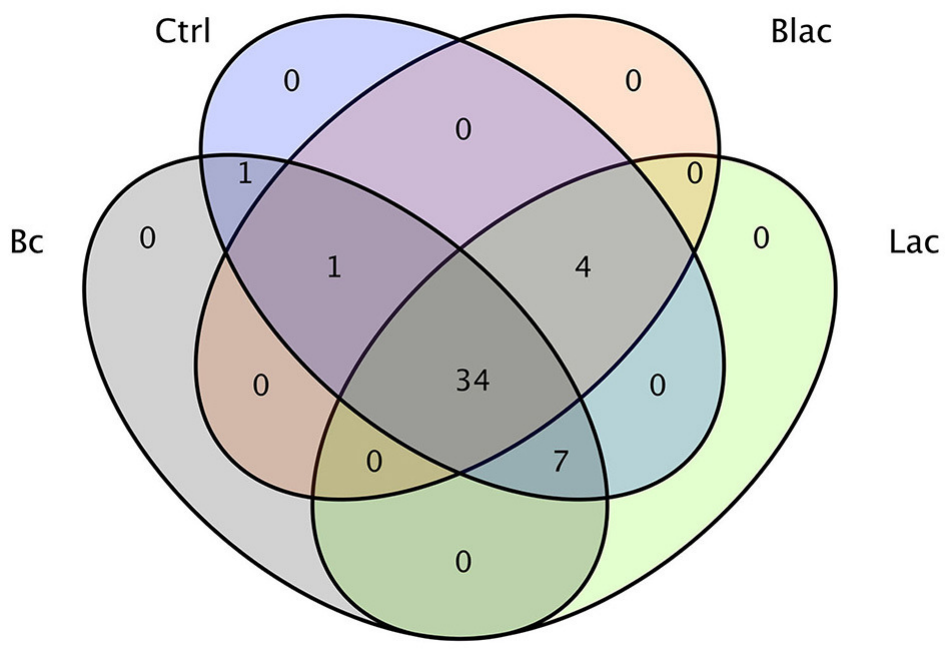
Operational taxonomic unit (OTU) Venn diagram of silage microorganism.

The results demonstrated a total of 47 OTUs in all samples, of which 34 were common to all the samples (Figure 3). There was one OTU shared between the Bc group and the Ctrl group; one OTU shared among the Bc, Blac, and Ctrl groups; four OTUs shared among the Ctrl, Blac, and Lac groups; and seven OTUs shared among the Bc, Lac, and Ctrl groups.

**Figure 4:**
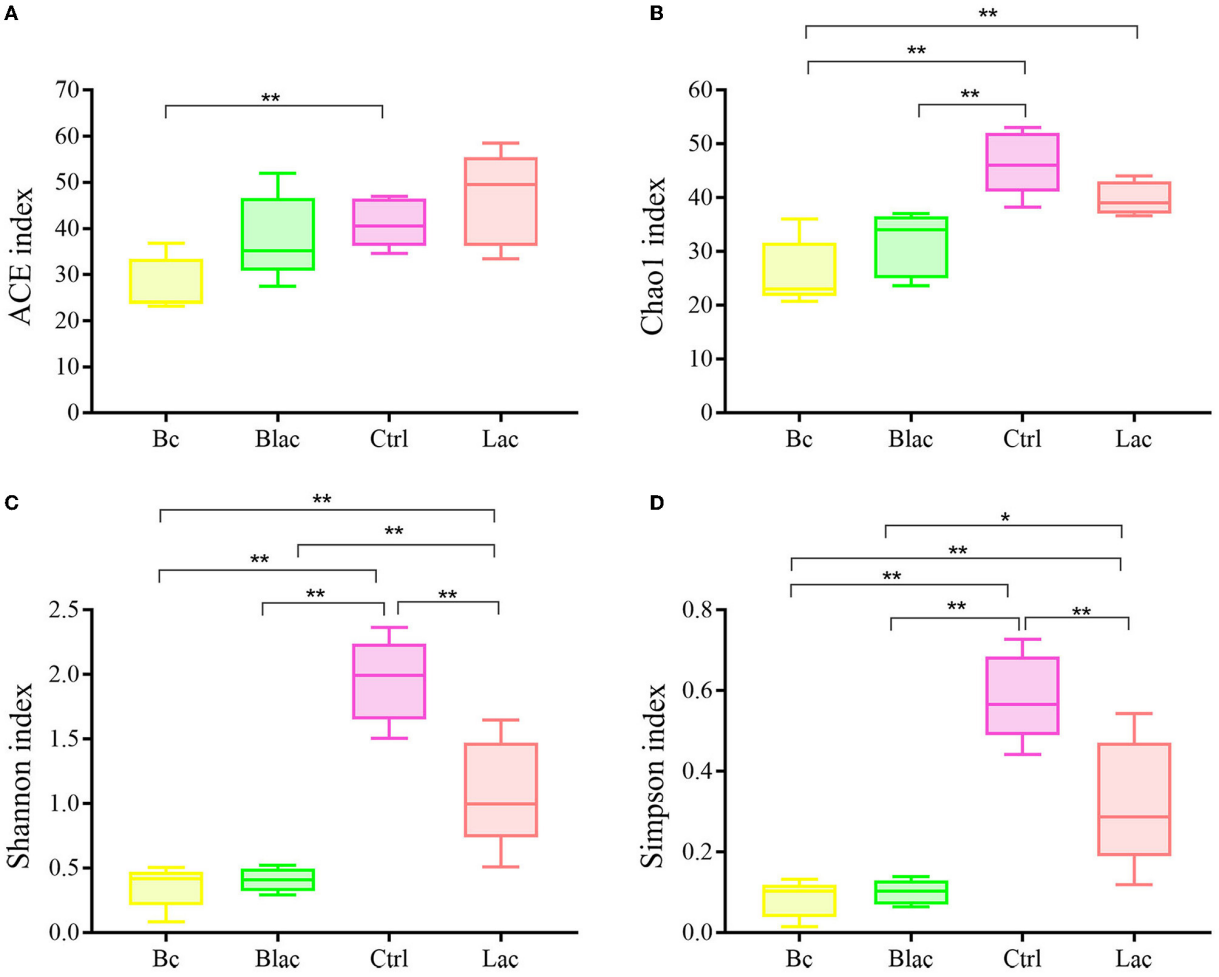
Alpha diversity indices [**(A)** Shannon index, **(B)** Ace index, **(C)** Chao1 index, and **(D)** Simpson index]; Bc, added with strain *B. cereus* AH7-7; Blac, added with strain *B. cereus* AH7-7, *Lactobacillus plantarum* and *Lactobacillus buchneri*; Lac, added with *Lactobacillus plantarum* and *Lactobacillus buchneri*; Ctrl, no additives. *means significant difference (*P* < 0.05); **means extremely significant difference (*P* < 0.01).

The Chao1 and ACE indices can reveal the richness of the bacterial communities, which are positively correlated with richness. The Shannon and Simpson indices can reveal the diversity of the bacterial communities, which are positively correlated with diversity. As shown in Figure 4, the ACE index of Bc group was significantly lower than that of the Ctrl group with no additive (*P* < 0.05). The Chao1 indices of the Bc group and the Blac group were significantly lower than those of the Ctrl group (*P* < 0.01). The Chao1 index of the Bc group was significantly lower than that of the Lac group (*P* < 0.01). The bacterial community richness of the Ctrl and Lac groups was significantly higher than the Bc and Blac groups. The Shannon and Simpson indices of Ctrl and Lac groups were significantly higher than those of the Bc and Blac groups (*P* < 0.01). The present result reflected a lower bacterial community diversity in the groups inoculated with *B. cereus* AH7-7.

**Figure 5:**
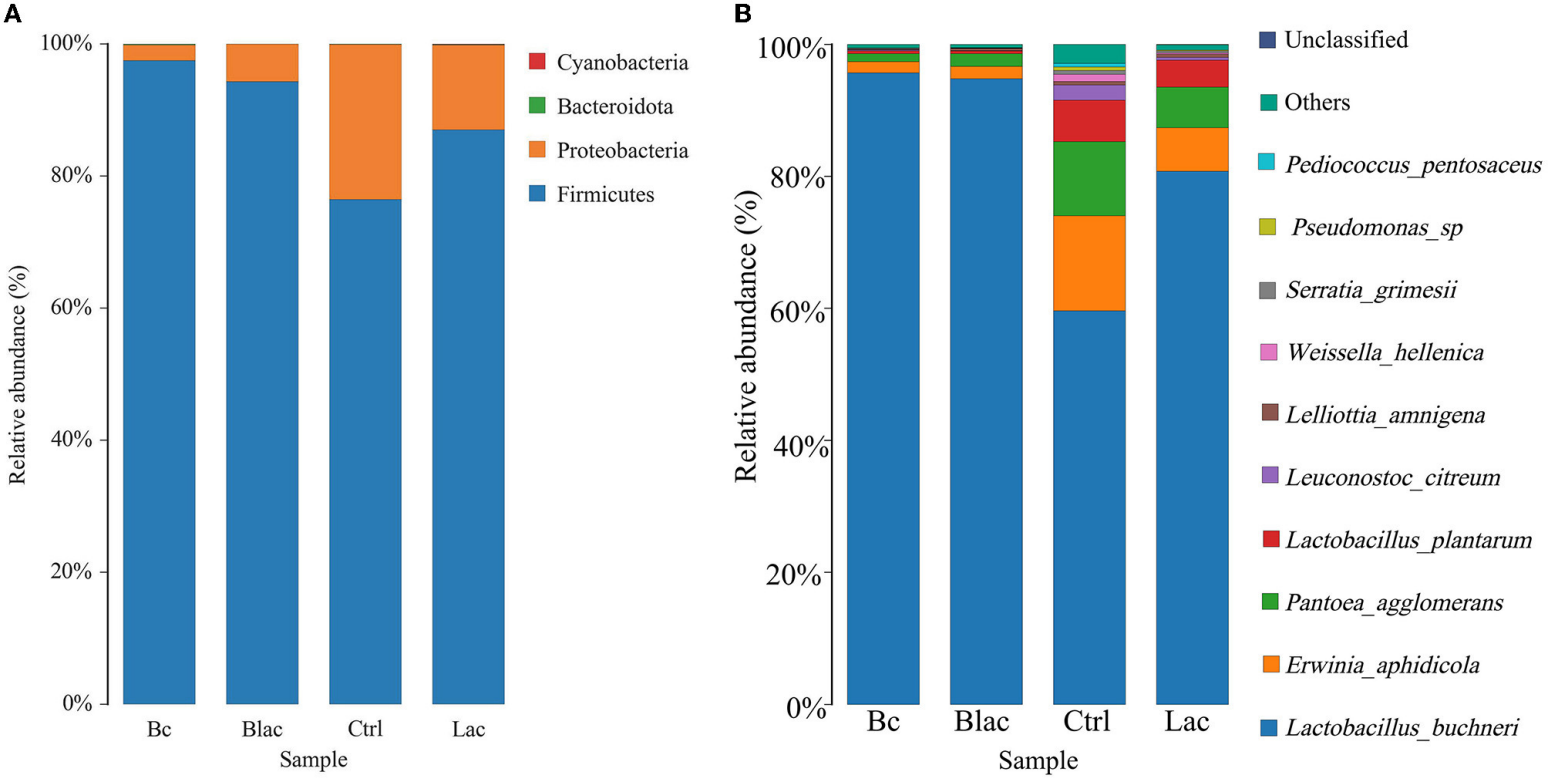
Distribution of microorganisms at the phylum level **(A)** and the species level **(B)**; Bc, added with strain *B. cereus* AH7-7; Blac, added with strain *B. cereus* AH7-7, *Lactobacillus plantarum* and *Lactobacillus buchneri*; Lac, added with *Lactobacillus plantarum* and *Lactobacillus buchneri*; Ctrl, no additives.

**Figure 6:**
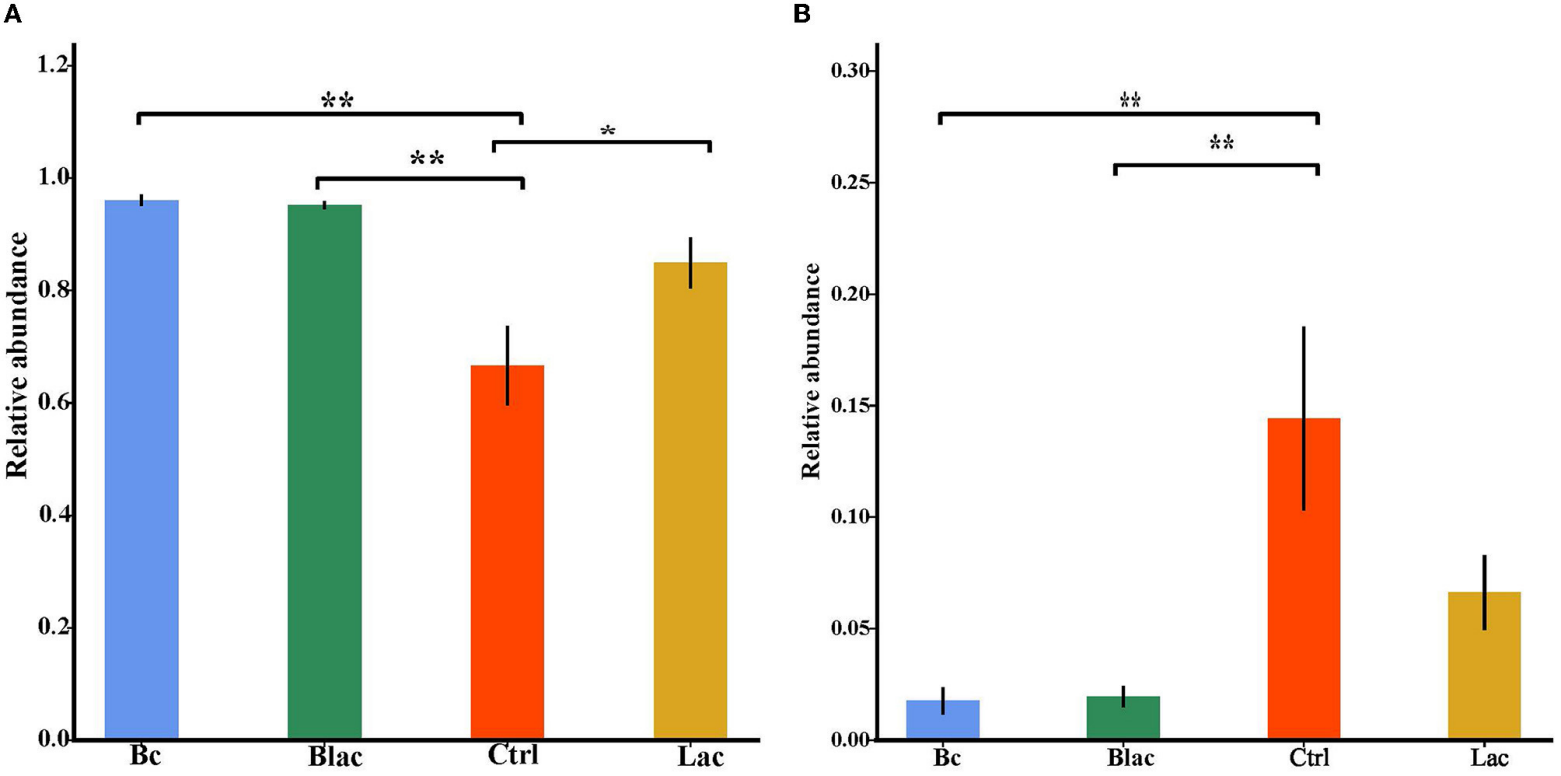
Histogram of analysis of variance among groups at *Lactobacillus* level **(A)** and *Erwinia* level **(B)**; *means significant difference (*P* < 0.05); **means extremely significant difference (*P* < 0.01); Bc, added with strain *B. cereus* AH7-7; Blac, added with strain *B. cereus* AH7-7, *Lactobacillus plantarum* and *Lactobacillus buchneri*; Lac, added with *Lactobacillus plantarum* and *Lactobacillus buchneri*; Ctrl, no additives.

As shown in Figure 5, the results revealed that Firmicutes and Proteobacteria were the main phyla of each group. Furthermore, the dominant species was *L. buchneri*. The relative abundance of *L. buchneri* in the Bc group was 95.67%, the Blac group was 94.76%, the Ctrl group was 59.64%, and the Lac group was 80.75%. The result of the analysis of variance indicated that the relative abundance of *Lactobacillus* in the groups inoculated with *B. cereus* AH7-7 (Bc and Blac) was higher (*P* < 0.01) (Figure 6A). The relative abundances of *Erwinia aphidicola* were 1.76%, 1.95%, 14.41%, and 6.63% in the Bc, Blac, Ctrl, and Lac groups, respectively, and the relative abundance of *Erwinia* was significantly higher in the Ctrl group (*P* < 0.01) (Figure 6B).

**Figure 7:**
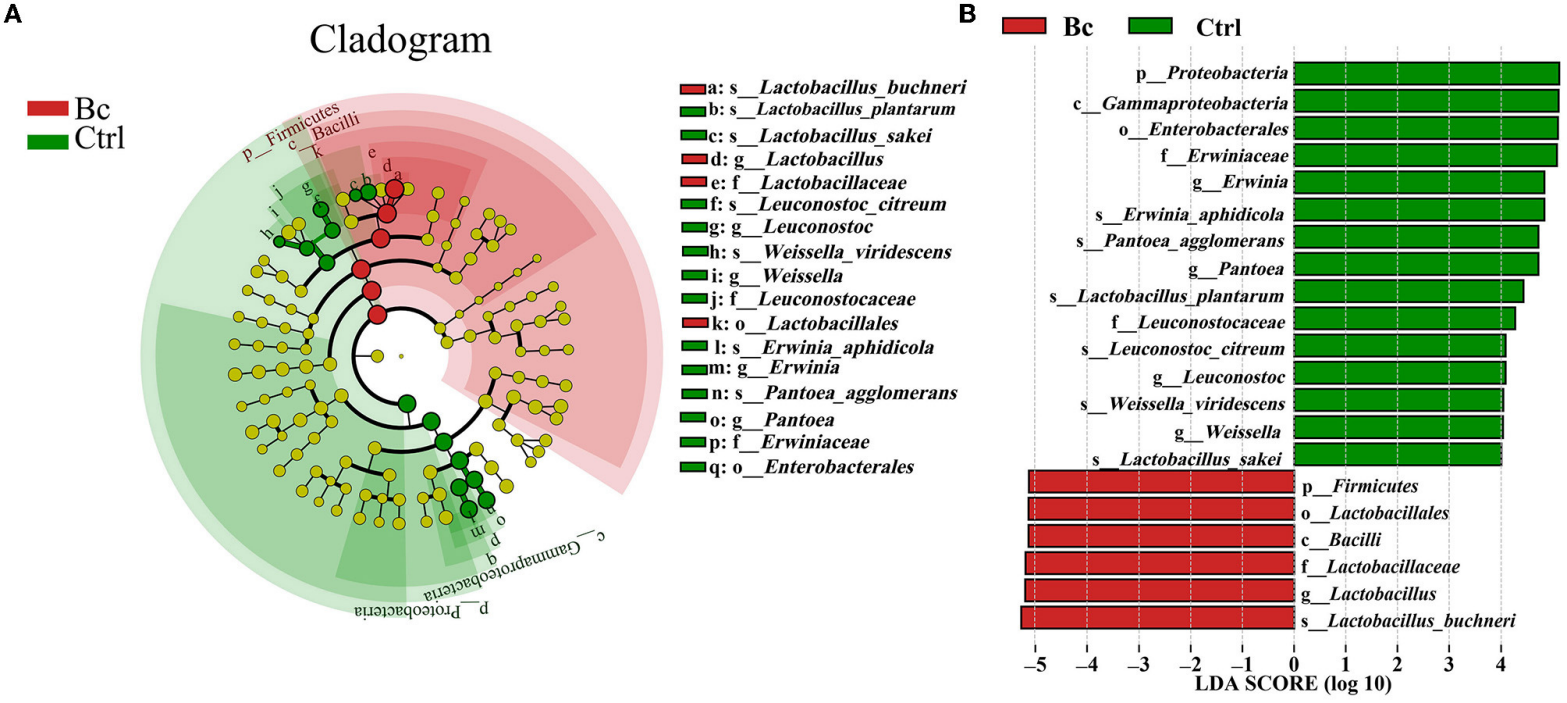
Line discriminant analysis (LDA) effect size. **(A)** Cladogram displays significantly enriched bacterial taxa (from the phylum to the species level). **(B)** Bar chart displays the LDA scores of the treatments. Significant differences are defined as *P* < 0.05 and LDA score >4.0. The classification level is from phylum to species. Bc, added with strain *B. cereus* AH7-7; Ctrl, no additives.

**Figure 8:**
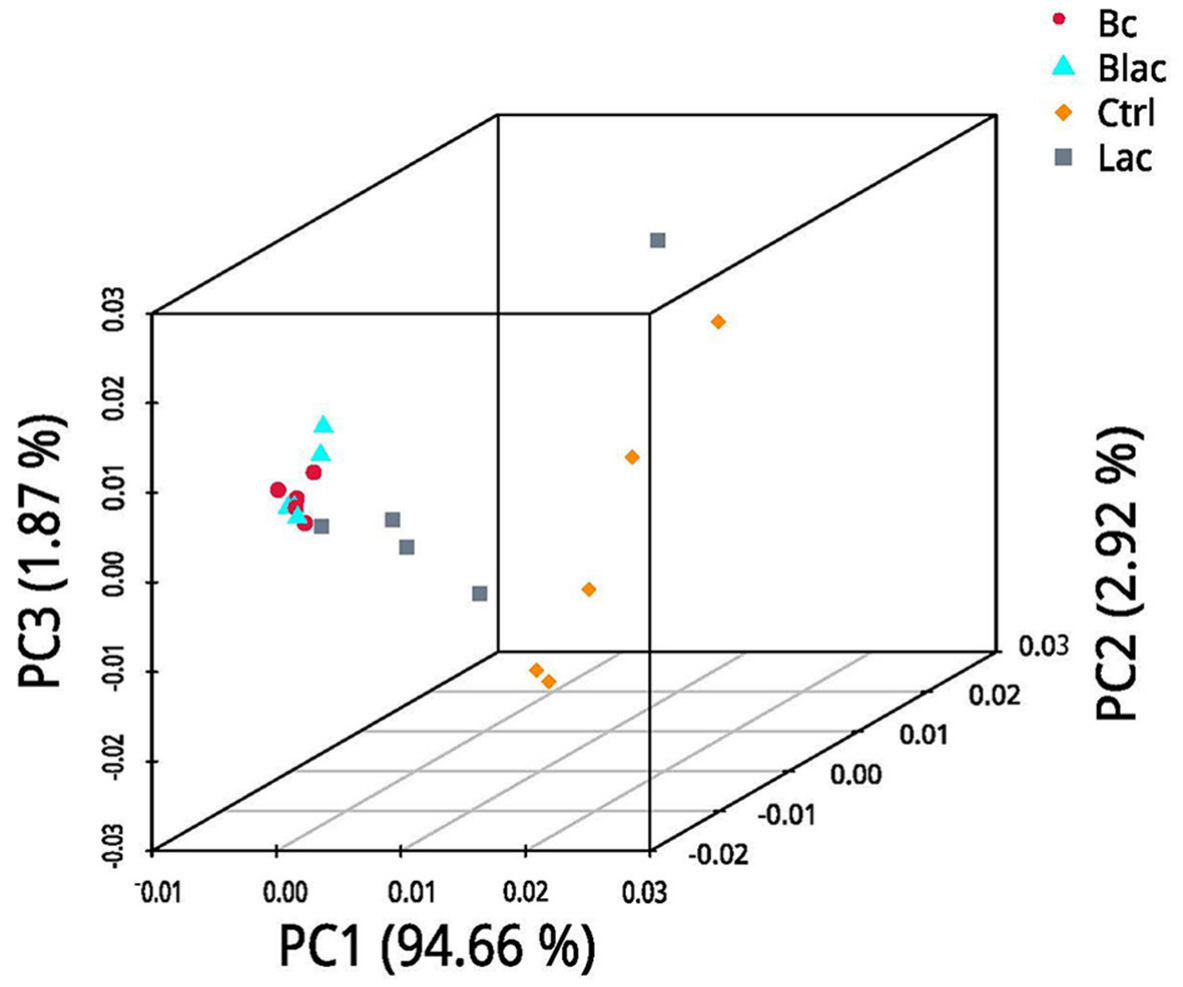
Principal component analysis (PCA); Bc, added with strain *B. cereus* AH7-7; Blac, added with strain *B. cereus* AH7-7, *Lactobacillus plantarum*, and *Lactobacillus buchneri*; Lac, added with *Lactobacillus plantarum* and *Lactobacillus buchneri*; Ctrl, no additives.

Under the LDA effect size analysis, there was no biomarker with a statistical difference between the Blac group and the Lac group, so the two groups are not shown in Figure 7. The cladogram (Figure 7A) shows that the level of *Lactobacillus* in the Bc group was dramatically higher than that in the Ctrl group. The levels of *Erwinia* and *Pantora* were enriched in the Ctrl group, whose abundances were higher than in the Bc group. The distribution histogram of an LDA value (Figure 7B) indicated that there were 21 kinds of biomarkers with a statistical difference in the Bc and Ctrl groups. Among them, 15 kinds of biomarkers were in the Ctrl group, and six were in the Bc group. The principal component analysis (PCA) result is shown in Figure 8, where principal components 1 (PC1), 2 (PC2), and 3 (PC3) explained 94.66%, 2.92%, and 1.87% of the total variance for the samples, respectively.

### 3.8. 16S rDNA gene-predicted functional profiles of rape silage after 60 days of ensiling

**Figure 9:**
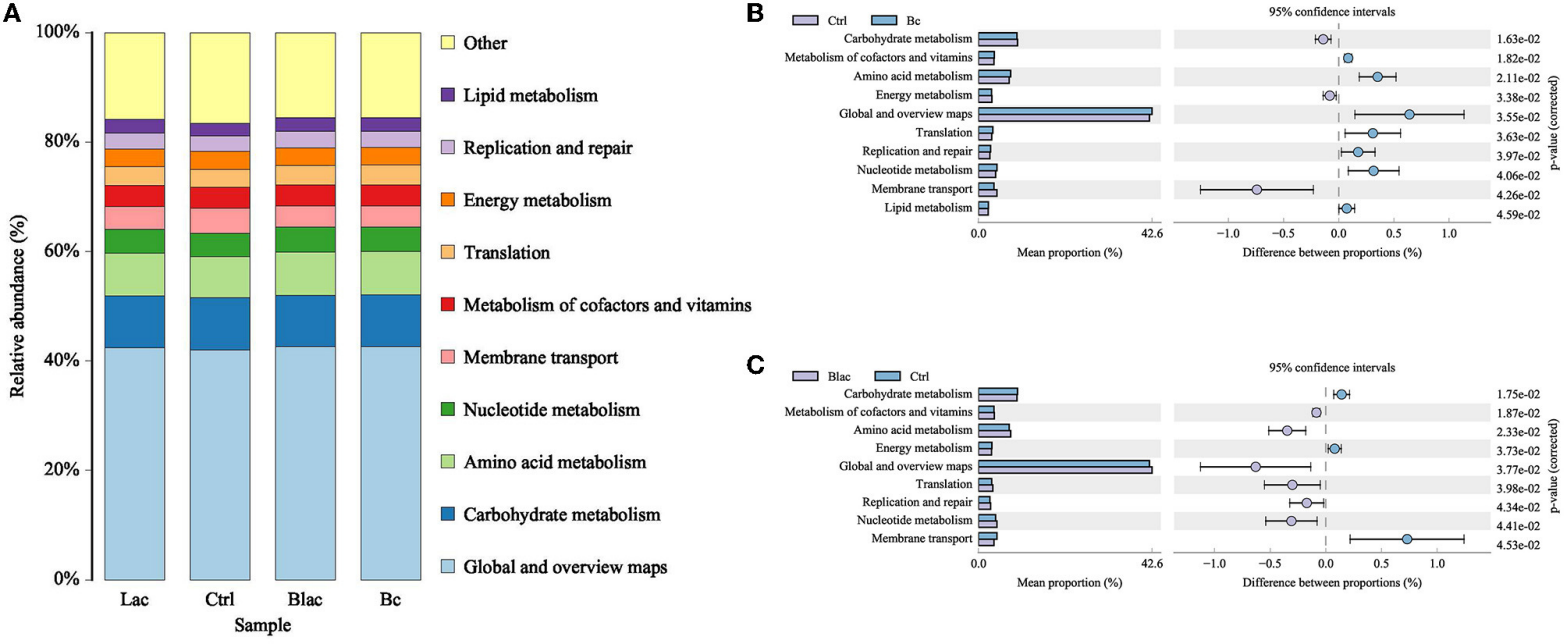
Histogram of function prediction results **(A)** and analysis of differences in metabolic pathways between Bc group and Ctrl group **(B)**, Blac group and Ctrl group **(C)**; Bc, added with strain *B. cereus* AH7-7; Blac, added with strain *B. cereus* AH7-7, *Lactobacillus plantarum*, and *Lactobacillus buchneri*; Lac, added with *Lactobacillus plantarum* and *Lactobacillus buchneri*; Ctrl, no additives.

The 16S rDNA gene-predicted functions of microbiota are shown in Figure 9. The main functions of each group were carbohydrate metabolism, amino acid metabolism, nucleotide metabolism, membrane transport, metabolism of cofactors and vitamins, translation, energy metabolism, replication and repair, and lipid metabolism. Compared with Ctrl group, the Bc and Blac groups, which inoculated with the *B. cereus* AH7-7 upregulated the metabolism of cofactors and vitamins, amino acid metabolism, translation, replication and repair, and nucleotide metabolism, and downregulated the carbohydrate metabolism, membrane transport, and energy metabolism. The Bc group increased lipid metabolism.

## 4. Discussion

### 4.1. Fermentation quality of rape silage

The pH value is an important indicator to assess the success of silage fermentation and to reflect microbial activity during ensiling (Kung et al., 2018). The pH values of the four study groups were lower than those of the previously described rape straw silage, regardless of whether *B. cereus* AH7-7 was added as a supplement (Li L. et al., 2019). The pH of Bc, Blac and Lac groups was in the range of good silage pH, while the pH of Ctrl group belonged to general silage pH (Abdelrahman et al., 2022). Lactic acid (LA) is the most important organic acid to rapidly lower silage pH (Kung et al., 2018). The lower pH level in the silage groups inoculated with *B. cereus* AH7-7 could be attributed to the increased LA formation by the additives. We speculated that the addition of *B. cereus* AH7-7 provided a beneficial fermentation environment for rape silage. Moreover, the low pH level may limit the growth of undesirable microorganisms because, on days 10, 30, and 60, no live *Enterobacteria* were detected in the silage.

The NH_3_-N level of silage is another direct index of silage quality (Hashemzadeh-Cigari et al., 2014). NH_3_-N represents the degree of proteolysis in the ensiling process, which is mainly from the plant enzymes that degrade proteins and the process of decomposition of proteins and amino acids by microorganisms (Wang et al., 2019). In the study, the groups that inoculated the exogenous strains as additives had lower NH_3_-N values. It demonstrated that the addition of exogenous strains can inhibit the activity of proteolytic bacteria, such as *Clostridial* (Wang et al., 2019). LA is the most important organic acid in the silage, which prevents the growth of spoilage microbes to minimize the loss of nutrients from the silage (Borreani et al., 2018). The water-soluble carbohydrate (WSC) content in fresh material affects the LA content during ensiling by promoting the fermentation of lactic acid bacteria (LAB) (Zeng et al., 2022). The ideal silage material should have a WSC content >50 g/kg dry matter (DM) (Li P. et al., 2019), and the rape material used in this research met this requirement with a WSC content of 72.43 g/kg DM. The result showed that the content of LA was increased when the *B. cereus* AH7-7 was added as the silage additive, especially in Blac group, indicating that the synergy of *B. cereus* AH7-7, *L. plantarum*, and *L. buchneri* produced greater effects on silage. The above results might be related to the higher WSC content in the group (Bc and Blac) inoculated with *B. cereus* AH7-7.

However, some anaerobic microorganisms may be present and decompose LA to produce acetic acid (AA) and propionic acid (PA), leading to a reduction in LA content (Shao et al., 2002). The AA in silage can effectively inhibit yeast growth, improving the stability of the ensiling process and reducing silage nutrient loss (Li and Nishino, 2013). *L. buchneri* is the main species to produce AA and PA in the silage (Krooneman et al., 2002; da Silva et al., 2018). The AA and PA contents were higher in the Bc and Blac groups, indicating the activity of *L. buchneri* during ensiling, which was supported by sequencing results indicating that the relative abundance of *L. buchneri* was higher in the groups inoculated with *B. cereus* AH7-7. The PA content at the end of the fermentation period of the groups (Bc and Blac) added with *B. cereus* AH7-7 was within the range of the high-quality silage (1–10 g/kg DM) (Wang et al., 2022a). Butyric acid (BA) reflects the nutrient loss in silage, and the better the fermentation, the less the BA (Wang et al., 2022b). Thus, compared to the Ctrl group, the groups with exogenous strain additives had a lower BA content, demonstrating that the addition of *B. cereus* AH7-7 could inhibit metabolic activity associated with the formation of BA.

### 4.2. Chemical composition of rape silage

The dry matter content and forage nutritional preservation are closely linked (Hu et al., 2009). The DM contents of the rape silage in this study ranged from 330.88 to 335.96 g/kg, which was higher than the DM contents of the rape straw silage studied by Li L. et al. (2019). Compared with the Ctrl group, the addition of *B. cereus* AH7-7 alone (Bc) could reduce the DM loss, and the effect was the same as that of the combination of *B. cereus* AH7-7, *L. plantarum*, and *L. buchneri* (Blac). Based on the results of pH values, we speculated that *B. cereus* AH7-7 could effectively promote pH reduction and reduce the consumption of DM by other microbes after its addition. Some reports on ruminal microorganisms applied to silage to reveal the effects of nutritional composition have been published (Lee et al., 2015; Li et al., 2018). Different from previous studies, *B. cereus* AH7-7 showed a unique characteristic in lignin-degradation ability. The degradation of structural carbohydrates during the ensiling to produce corresponding sugars could be attributed to acidolysis, enzymatic action, and microbial activity (Dewar et al., 1963). Lignin fills in the gap between cellulose and hemicellulose to limit the utilization of cellulose and hemicellulose (Zakzeski et al., 2010; Zhong et al., 2021). *B. cereus* AH7-7 could degrade lignin and expose readily hydrolyzable fiber fractions in silage, which were hydrolyzed by organic acids to yield more sugars. That might be the reason why the contents of WSC were higher in the groups that were inoculated with *B. cereus* AH7-7. Notably, the Blac group, which was inoculated with *B. cereus* AH7-7, *L. plantarum*, and *L. buchneri* additives, had the highest WSC level. This demonstrated that the synergy among bacteria might promote the production of WSC. Another reason might be that the lignin degradation reaction accelerated oxygen consumption, thereby inhibiting the activity of aerobic microorganisms and saving more fermentable sugars. The reduction of cellulose and hemicellulose contents and the increase of WSC contents in the Bc and Blac groups supported the above partially held view. The results showed that the addition of exogenous strains inhibited the protein hydrolysis of rape silage and improved the crude protein content of rape silage, especially in the Bc and Blac groups. This seems to be due to the fact that treatments with additives accelerated the decline of pH values and limited the hydrolysis processes of plant and microbial proteins. *B. cereus* AH7-7 has been proven to have the encoded gene for laccase (Zhong et al., 2022). The result was consistent with the previous report that laccase could improve the true protein content in silage (Bao et al., 2022). As expected, *B. cereus* AH7-7 degraded lignin in silage, resulting in a significant reduction in acid detergent lignin (ADL) content in groups supplemented with *B. cereus* AH7-7. Adding laccase before ensiling could reduce ADL content (Bao et al., 2022), and we speculated that the laccase produced by *B. cereus* AH7-7 promoted the degradation.

### 4.3. The microbial population of rape silage

The microbial population is closely related to the quality and microbial community of silage. Many LAB have been reported to improve the ensiling process, such as *L. buchneri* (Kung and Ranjit, 2001), *L. plantarum* (Xu et al., 2019), and *Weissella* (Ammor and Mayo, 2007), which produce quantities of LA in a short time, lower the pH, reduce the nutrition loss, and inhibit the growth of undesirable microorganisms, ultimately leading to high-quality silage (Weinberg and Ashbell, 2003; Ni et al., 2015). Previous studies indicated that the number of LAB on fresh materials should not be <5.00 log_10_ CFU/g FW (Wang et al., 2017). The number of LAB on fresh materials in this experiment met this requirement, which was 5.26 log_10_ CFU/g FW LAB in rape. During the ensiling period, the number of LAB in each group increased with time and was the highest at the end of fermentation (60 days). If a large number of undesirable microorganisms (>6.54 log_10_ CFU/g FW), such as yeasts and *Enterobacter*, are present in fresh rape, this might lead to poor fermentation quality. Yeast is an aerobic microbial species that competes with LAB for WSC (Spoelstra et al., 1988; Lv et al., 2020). In this study, the yeasts were found in the groups without *B. cereus* AH7-7 additives (Ctrl and Lac) after 10 days of ensiling, which might be one of the reasons affecting the fermentation quality and chemical compositions of silage at the end of fermentation. In silage, AA and PA have an antifungal effect. *L. buchneri* is the main species that produces AA and PA. The presence of yeasts in the Lac and Ctrl groups after 10 days of ensiling might be associated with low *L. buchneri* abundance and low levels of AA and PA. Additionally, the molds and *Enterobacter* were not detected after 10, 30, or 60 days of ensiling in this study. The low pH of rape silage might inhibit the growth of these microbes.

### 4.4. Bacterial diversity and composition of rape silage

The alpha diversity is used to estimate the richness, diversity, and evenness of species in bacterial communities (Chi et al., 2022). According to the Good's coverage values (> 99%) in all groups, the sequencing analysis sufficiently represented the real situation for the bacterial community of rape silage. In this study, the bacterial diversity was reduced in the *B. cereus* AH7-7 additive-treated silage groups compared with the no *B. cereus* AH7-7 additive groups. An established view is that microbial diversity is sharply reduced after ensilage, and the LAB occupy a dominant position. Ren indicated that the high quality of silage usually correlates with a lower microbial diversity (Ren et al., 2019). The decrease of bacterial diversity in the Bc and Blac groups may be due to the addition of *B. cereus* AH7-7, which accelerated the decline of pH values and inhibited the growth of other undesirable microorganisms. Furthermore, the PCA analysis also indicated that *B. cereus* AH7-7 could limit the growth of other microbes. The results showed that the *B. cereus* AH7-7 additive treatments reshaped the structure of the bacterial community of rape silage. The samples in the groups without adding *B. cereus* AH7-7 have greater differences. On the contrary, the microbial composition of the groups with *B. cereus* AH7-7 additive was more similar.

It has been proven that microbial additives can affect the quality of silage by changing the bacterial composition (Ni et al., 2017; Liu et al., 2019). In this experiment, the bacterial compositions of different silage groups at the phylum level were relatively similar, involving mainly Firmicutes. The results were consistent with previous studies on successful grass ensiling (Eikmeyer et al., 2013; Jiang et al., 2020). Ren also concluded that high-quality silage usually has fewer shared and unique bacterial OTUs (Ren et al., 2019). In this study, there were only 47 OTUs in total in the four groups. Compared with the treatments without *B. cereus* AH7-7, there were fewer OTUs in the groups Bc and Blac, which were added with *B. cereus* AH7-7. The addition of *B. cereus* AH7-7 might reduce OTUs and simplify the microbial composition of silage by inhibiting other microorganisms. LAB are common additives applied as stimulants to promote silage fermentation (Muck et al., 2018), which can improve livestock performance by inhibiting detrimental microbes and interacting with rumen microbes (Weinberg and Ashbell, 2003; Ellis et al., 2016). LAB promote the effective preservation of silage by producing LA (Blajman et al., 2018). After successful silage, the abundance of LAB expands, and LAB occupy the whole biota and become the dominant group (Guan et al., 2020), which is a key factor in evaluating the silage. Unexpectedly, the group inoculated with *B. cereus* AH7-7 alone and the group that applied the combination of *B. cereus* AH7-7, *L. plantarum*, and *L. buchneri* additives exhibited a dramatic *Lactobacillus* richness, probably because *B. cereus* AH7-7 inhibited the undesired bacteria, creating optimal conditions for the rapid growth of LAB. This observation was also confirmed by the alpha diversity analysis; the group with *B. cereus* AH7-7 additive alone and the group with the combination of *B. cereus* AH7-7, *L. plantarum*, and *L. buchneri* had the lowest microbial diversity. In addition to *Lactobacilli, Weissella, Pediococcus, and Leuconostoc* were also identified as LA production strains, which are known to function as the early initiators of silage LA fermentation but would be gradually outcompeted by *Lactobacillus* species as fermentation progressed (Cai et al., 1999). The abundance of the above strains was lower in the Bc and Blac groups, also showing that the *B. cereus* AH7-7 additive improved the advantage of *Lactobacillus*. Conversely, undesirable strains, such as *Pantoea*, which is a genus separated from the genus *Enterobacter*, compete with LAB for nutrients and increase BA content in silage (Lianhua et al., 2017). *Erwinia* has been reported to be a plant parasite that causes putrefaction (Pérombelon, 2002; Toaza et al., 2021), which can deteriorate the silage quality and affect the production of livestock (Flythe and Russell, 2004). Interestingly, according to the sequencing result, we found that the abundance of *Erwinia* and *Pantoea* in the group with no additive was higher than in the groups added with *B. cereus* AH7-7. Consequently, *B. cereus* AH7-7 efficiently reduces the pernicious strains in the silage process.

### 4.5. Functional profiles of rape silage

Predicting the functional profiles of the bacterial community contributes to evaluating the quality of silage. The main functions of each group were carbohydrate metabolism, amino acid metabolism, nucleotide metabolism, membrane transport, and metabolism of cofactors and vitamins, which were consistent with past reports (Wang et al., 2022a). As essential substances for plants, amino acids are essential for promoting primary metabolism and plant protein synthesis. Herein, the relative abundance of amino acid metabolism was higher in the *B. cereus* AH7-7 treated groups than the Ctrl group, the same as in the previous report that the silage with laccase additive (Bai et al., 2021). This phenomenon might be related to the increased LA content in the groups added with *B. cereus* AH7-7, as LA formation involves amino acid decarboxylation and arginine deamination (Bai et al., 2021).

Carbohydrate metabolism mainly contained gluconeogenesis and glycolysis metabolism (Kanehisa and Goto, 2000). In this study, the groups inoculated with *B. cereus* AH7-7 showed lower levels of carbohydrate metabolism, possibly due to lower bacterial diversity, which contributed to the carbohydrate metabolism process being mainly concentrated in LAB using WSC fermentation to produce LA, reducing the sugar fermentation process of other undesirable microorganisms. Higher LA content and LAB levels in the BC and Blac groups confirmed this result. The present research showed that the pathways of nucleotide metabolism, translation, and replication and repair were increased in the groups inoculated with *B. cereus* AH7-7. These increased genetic functions in the *B. cereus* AH7-7 treated silages were probably due to the existence of high-activity LAB. Previous studies found that the use of LAB additives can improve the yield of vitamins in silage (Zhang et al., 2020; Bai et al., 2021). *B. cereus* AH7-7 might play a similar role in silage, increasing the metabolism of cofactors and vitamins. In addition, compared with the Ctrl group, the relative abundance of energy metabolism and membrane transport was decreased in the groups inoculated with *B. cereus* AH7-7. The group added *B. cereus* AH7-7 alone, which increased lipid metabolism. *B. cereus* AH7-7 has the ability to degrade lignin, but we do not know the specific mechanism of its action in silage, which might mobilize metabolism routes linking to amino acids, lipids, and energy (He et al., 2021). Many phenomena still cannot be explained accurately. Hence, it is necessary to use some omics methods, such as transcriptome, metabolome, and proteomics, to further investigate the function of bacterial communities during ensiling.

## 5. Conclusion

A strain of *B. cereus* AH7-7 with lignin degradation ability and acid tolerance was screened and identified from buffalo rumen. The *B. cereus* AH7-7 enhanced fermentation quality and decreased fermentation loss of silage when inoculated in rape before ensiling. The contents of lactic acid, protein, and water-soluble carbohydrates were increased, and the contents of NH_3_-N, butyric acid, and acid detergent lignin were decreased in groups inoculated with *B. cereus* AH7-7. Meanwhile, the bacterial diversity of rape silage decreased, the relative abundance of *Lactobacillus* increased, and that of *Pantoea* and *Erwinia* decreased. The bacterial metabolic pathways in silage were mainly related to cofactors and vitamin metabolism, amino acid metabolism, and nucleotide metabolism. *B. cereus* AH7-7 inoculant could improve the fermentation quality, nutrient preservation, and bacterial community of rape silage, especially with the combination and synergy of *B. cereus* AH7-7, *L. plantarum*, and *L. buchneri*. The *B. cereus* AH7-7 additive can be a promising strategy for improving silage quality.

## Data availability statement

The datasets presented in this study can be found in online repositories. The names of the repository/repositories and accession number(s) can be found below: https://www.ncbi.nlm.nih.gov/, SRR17968813 https://www.ncbi.nlm.nih.gov/, SRR17968814 https://www.ncbi.nlm.nih.gov/, SRR17968815 https://www.ncbi.nlm.nih.gov/, SRR17968816 https://www.ncbi.nlm.nih.gov/, SRR17968817 https://www.ncbi.nlm.nih.gov/, SRR17968818 https://www.ncbi.nlm.nih.gov/, SRR17968819 https://www.ncbi.nlm.nih.gov/, SRR17968820 https://www.ncbi.nlm.nih.gov/, SRR17968821 https://www.ncbi.nlm.nih.gov/, SRR17968822 https://www.ncbi.nlm.nih.gov/, SRR17968823 https://www.ncbi.nlm.nih.gov/, SRR17968824 https://www.ncbi.nlm.nih.gov/, SRR17968825 https://www.ncbi.nlm.nih.gov/, SRR17968826 https://www.ncbi.nlm.nih.gov/, SRR17968827 https://www.ncbi.nlm.nih.gov/, SRR17968828 https://www.ncbi.nlm.nih.gov/, SRR17968829 https://www.ncbi.nlm.nih.gov/, SRR17968830 https://www.ncbi.nlm.nih.gov/, SRR17968831 https://www.ncbi.nlm.nih.gov/, SRR17968832.

## Ethics statement

The animal study was reviewed and approved by Animal Experimental Ethical Inspection of Laboratory Animal Center, Huazhong Agriculture University (HZAUCA-2018-003). Written informed consent was obtained from the owners for the participation of their animals in this study.

## Author contributions

HZ and XL conceived and designed the study. HZ, JZ, FW, and WW performed research. HZ and JZ extracted and analyzed the data. HZ wrote the manuscript. HZ, JZ, and XL revised the manuscript. HX and HY provided rape raw materials. All authors read and approved the final manuscript.

## Supplementary material

Supplementary Figure 1 **(A)** Observation of colonies from strain AH7-7. **(B)** Gram staining of the strain AH7-7 under the microscope (magnification 1,000 × , Olympus BX53).

Supplementary Figure 2 Acid tolerance of the strain AH7-7 to different pHs.

Supplementary Table 1 Results of physiological and biochemical characteristics of the strain AH7-7 in the API20E identification system.

Supplementary Table 2 Results of physiological and biochemical characteristics of the strain AH7-7 in the API50CH identification system.

Supplementary Table 3 Blood routine, DM feed intake, and body condition scores of buffalo.
