# Supplementary material for: Isolation and identification of ligninolytic bacterium (Bacillus cereus) from buffalo (Bubalus bubalis) rumen and its effects on the fermentation quality, nutrient composition, and bacterial community of rape silage
Source: Front Microbiol. 2023 Apr 18;14:1103652. doi: 10.3389/fmicb.2023.1103652 (PMC10153755; doi:10.3389/fmicb.2023.1103652)
Supplement: Supplementary Figure 1 — (A) Observation of colonies from strain AH7-7. (B) Gram staining of the strain AH7-7 under the microscope (magnification 1,000 × , Olympus BX53). [file Data_Sheet_1.docx]

Supplementary Material

## Supplementary Figures


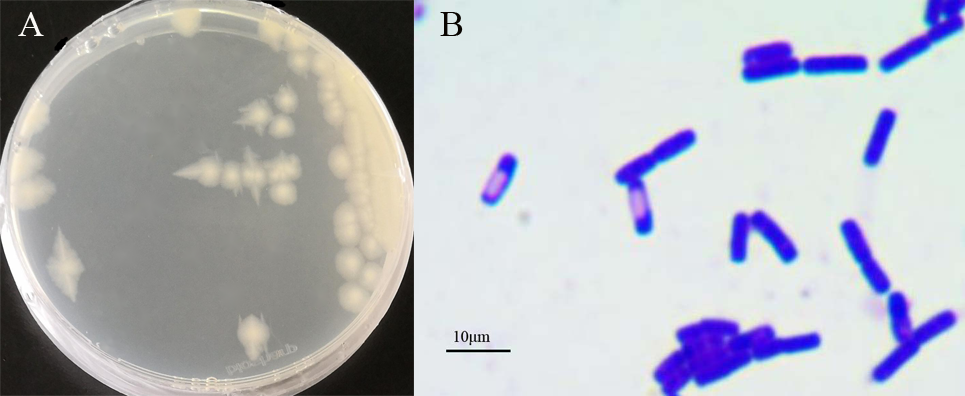


**Supplementary Figure 1.** **(A)** Observation of colonies from strain AH7-7. **(B)** Gram's staining of strain AH7-7 under the microscope (magnification 1000×, Olympus BX53).

**
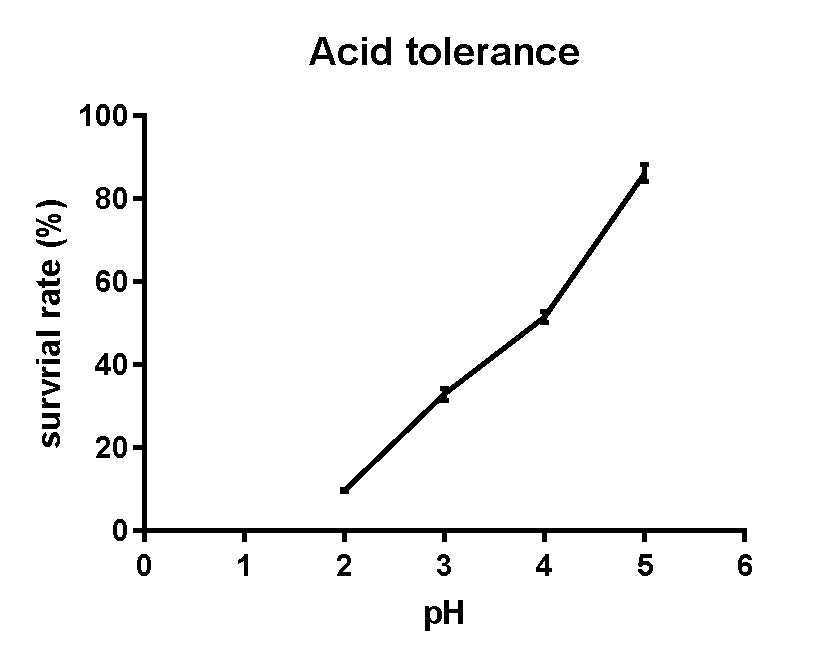
**

**Supplementary Figure 2.** **Acid tolerance of strain AH7-7 to different pH**

## Supplementary Tables

**Supplementary Table 1.** The results of physiological and biochemical characteristics of strain AH7-7 in API20E identification system

| **Substrate** | **Fermentation/oxidation** | **Enzyme activity** | **Results** | **Substrate utilisation** | **Results** |
| --- | --- | --- | --- | --- | --- |
| Glucose | — | β-Galactosidase | — | Citric acid utilisation | + |
| Mannitol | — | Arginine bishydrolase | + | Production of H_2_S | — |
| Inose | — | Lysine decarboxylase | — | Production of indole | — |
| Sorbic alcohol | — | Ornithine decarboxylase | — | Production of acetylmethyl methanol | w |
| Rhamnose | — | Urease | — |  |  |
| Saccharose | — | Tryptophan deaminase | — |  |  |
| Melibiose | — | Gelatinase | + |  |  |
| Amygdalin | — |  |  |  |  |
| Arabinose | — |  |  |  |  |

Note: + means positive reaction, — means negative reaction, w means weakly positive reaction.

**Supplementary Table 2.** The results of physiological and biochemical characteristics of strain AH7-7 in API50CH identification system

| **Substrate** | **Results** | **Substrate** | **Results** | **Substrate** | **Results** |
| --- | --- | --- | --- | --- | --- |
| Control | — | Urosside | + | Glucose | + |
| Glycerol | — | Aesculin | + | Fructose | + |
| Erythritol | — | Salicin | + | Seminose | — |
| D-Arabinose | — | Cellobiose | — | Sorbose | — |
| L-Arabinose | — | Maltose | + | Rhamnose | — |
| Ribose | w | Lactose | — | Melampyrin | — |
| D-Xylose | — | Melibiose | — | Increositol | — |
| L-Xylose | — | Saccharose | + | Mannitol | — |
| Adonite | — | Trehalose | + | Sorbic alcohol | — |
| Galactose | — | Inulin | — | α-Methyl-D-mannoside | — |
| D-Arabinol | — | Melezitose | — | α-Methyl-d-glucoside | — |
| L-Arabinol | — | Raffinose | — | N-Acetyl-glucosamine | — |
| Amygdalin | — | Starch | — | 2-Keto-gluconate | — |
| Gluconate | — | Glycogen | + | 5-keto-gluconate | — |
| D-Turanose | — | Xylosic alcohol | — | β-Methyl-D-xyloside | — |
| D-Tagatose | — | D-Lyxose | — |  |  |

Note: + means positive reaction, — means negative reaction, w means weakly positive reaction.

**Supplementary Table 3. Blood routine, DM intake and body condition score of buffalo.**

| **Items** | **buffalo 1** | **buffalo 2** | **buffalo 3** | **Reference value** |
| --- | --- | --- | --- | --- |
| RBC (10^12^/L) | 7.40 | 8.53 | 6.18 | 5.0-10 |
| HGB (g/dL) | 12.70 | 9.60 | 12.60 | 8-15 |
| MCV (fL) | 41.00 | 42.00 | 45.00 | 40-60 |
| MCH (pg) | 12.80 | 11.60 | 13.10 | 11-17 |
| MCHC (g/dL) | 33.00 | 33.80 | 35.30 | 30-36 |
| WBC (10^9^/L) | 6.55 | 4.87 | 10.63 | 4-12 |
| LY (10^9^/L) | 7.40 | 5.16 | 6.57 | 2.5-7.5 |
| MO (10^9^/L) | 0.56 | 0.44 | 0.48 | 0-0.84 |
| **Items** | **buffalo 1** | **buffalo 2** | **buffalo 3** | **SEM** |
| DM intake (kg/d) | 8.97 | 9.23 | 10.53 | 0.29 |
| Body condition score | 3.25 | 3.00 | 3.75 | 0.12 |

Note: RBC (red blood cell). HGB (haemoglobin). MCV (mean corpuscular volume). MCH (mean corpuscular haemoglobin). MCHC (mean haemoglobin concentration). WBC (white blood cell). LY (lymphocyte). MO (monocyte). SEM (standard error of mean).
